# Enrichment in Antioxidant Flavonoids of Stamen Extracts from *Nymphaea lotus* L. Using Ultrasonic-Assisted Extraction and Macroporous Resin Adsorption

**DOI:** 10.3390/antiox9070576

**Published:** 2020-07-02

**Authors:** Duangjai Tungmunnithum, Samantha Drouet, Atul Kabra, Christophe Hano

**Affiliations:** 1 Department of Pharmaceutical Botany, Faculty of Pharmacy, Mahidol University, Bangkok 10400, Thailand; 2 Laboratoire de Biologie des Ligneux et des Grandes Cultures, INRA USC1328, University of Orleans, CEDEX 2, 45067 Orléans, France; samantha.drouet@univ-orleans.fr; 3 Bioactifs et Cosmetiques, CNRS GDR 3711 Orleans, CEDEX 2, 45067 Orléans, France; 4 School of Pharmacy, Raffles University, Neemrana 301705, Alwar, Rajasthan, India; dr.atulkabra@rafflesuniversity.edu.in

**Keywords:** *Nymphaea lotus* L., stamen, ultrasound-assisted extraction (UAE), macroporous resin (MPR) purification, in vitro, *in cellulo*, antioxidant, flavonoids

## Abstract

*Nymphaea lotus* L. is the medicinal plant that has long been used for food, cosmetics and traditional medicines in Africa and Asia since ancient times. Its flavonoids and other interesting phytochemical compounds from rhizome, leaf and the whole flowers have been reported in the previous published research. However, stamens, which are essential for reproductive functions, may also represent new alternative sources of potential antioxidant flavonoids, as investigated in this study. The innovative green chemistry methods, i.e., ultrasound-assisted extraction (UAE) as well as a macroporous resin (MPR) purification procedure, were employed in this current research. Using a full factorial design coupled to three-dimensional (3D) surface plot methodology, the influence of three variables, namely aqEtOH concentration (ranging from 50 to 100% (*v*/*v*), US frequency (ranging from 0 (no US applied) to 45 kHz), and the extraction duration (ranging from 20 to 60 min), were evaluated. Five MPRs with different surface areas, average pore diameters, matrix types and polarities were also investigated for the purification of total flavonoids. The optimal UAE condition is 90% (*v*/*v*) aqEtOH with 34.65 khz ultrasonic frequency and 46 min of extraction duration. Compared with the conventional heat reflux extraction (HRE) method, a significant 1.35-fold increase in total flavonoids content was obtained using optimized UAE conditions (169.64 for HRE vs. 235.45 mg/g dry weight for UAE), causing a 2.80-fold increase when this UAE associated with MPR purification (475.42 mg/g dry weight). In vitro cell free antioxidant activity of *N. lotus* stamen extracts and in cellulo antioxidant investigation using yeast model showed the same trend, indicating that the best antioxidant flavonoid can be found in UAE coupled with MPR purification. Moreover, in the yeast model, the expression of key antioxidant genes such as *SIR2* and *SOD2* were expressed at the highest level in yeast cells treated with the extract from UAE together with MPR purification. Consequently, it can be seen that the UAE combined with MPR purification can help enhance the flavonoid antioxidant potential of the stamens extract from this medicinal species.

## 1. Introduction

**Figure 1:**
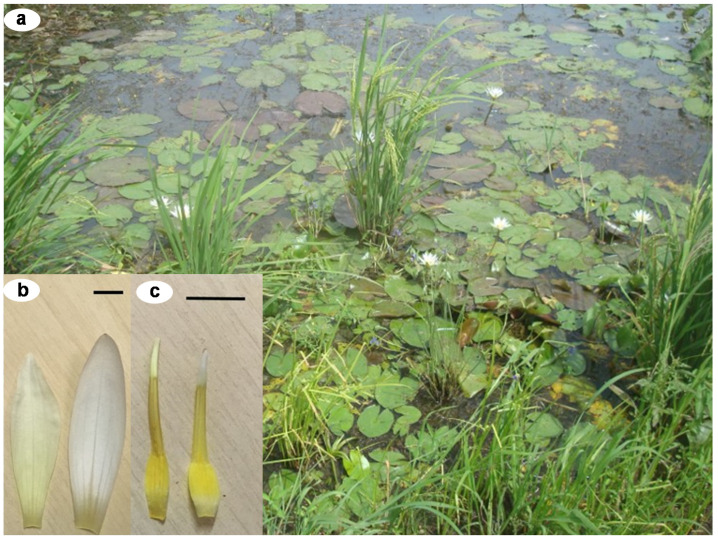
Pictures of *Nymphaea lotus* plants used in the present study. (**a**) *N. lotus* in its natural habitat in Nakhon Sawan province, Northern Floristic Regions of Thailand; (**b**) *N. lotus* petals (Bar scale = 1 cm); (**c**) *N. lotus* stamens (Bar scale = 1 cm). Pictures by Duangjai Tungmunnithum.

*Nymphaea lotus* L. (Figure 1) is commonly known as the water lily or Egyptian lotus. This medicinal plant is native to Africa, Asia and some specific areas in Europe. It has various vernacular names depending on the country, e.g., Bau-Sai (Thailand), Nettai Suiren (Japan), Sulyeon (Korea) and Bashneen Abiad (Egypt). *N. lotus* is an aquatic flowering herb with tuberous rhizome. Due to the beauty of its flower, this plant is widely used and cultivated as an ornamental plant. Moreover, *N. lotus* is used as a medicinal plant for traditional medicines, food and herbal tea for health benefits, especially in many Asian countries, Egypt and other countries in Africa [1,2,3,4,5]. Almost every part of this medicinal plant is edible. Its petiole and peduncle can be eaten raw as a vegetable or cooked with meats for curry menu; its flower and peduncle are popular in some traditional desserts [3,6,7,8,9].

In the past few decades, this medicinal plant has received continuously growing interested for its economical and pharmacological potentials. Many research teams have conducted studies focusing on *N. lotus* in several aspects. The safety of *N. lotus* flower extracts was verified by Kameni et al. [10]. Acute, sub-chronic and neurotoxicity were studied in vivo using an albinos Wistar rat model. This study indicated that the *N. lotus* flowers extract probably account for neuroprotective, immune-boosting and antioxidant activity without neurotoxicity [10]. Furthermore, the pharmacological and medicinal potential of this medicinal species were currently confirmed at in vivo level, i.e., anti-diarrhea effect of *N. lotus* rhizome extract [5], anxiolytic and antidepressant potential from the *N. lotus* leaf extract [11]. Oyeyemi et al. [12] investigated the hepatoprotective effect of *N. lotus* methanol extract to fight against carbon tetrachloride-induced chronic hepatotoxicity in vivo model using Wistar rats. Their results indicated that extracts of *N. lotus* show hepatoprotective potential through an antioxidative mechanism [12]. The strong antioxidant activity of extract of *N. lotus* flower compared to other medicinal plants was reported and related to their flavonoid content [13]. *N. lotus* is known to accumulate various flavonol glycosides in both vegetative and reproductive parts [4,14,15]. In particular, flavonoids composition has been related to flower color variations of *Nymphaea* species [14].

The development of effective extraction methods is important for the optimal valuation of plant extracts. Different methods have been developed for the extraction of natural antioxidants from various natural matrices based on traditional methods, such as maceration or extraction by Soxhlet. More recently, green extraction methods for plant-natural products, such as microwave-assisted [16], enzymatic-assisted [17] or ultrasound-assisted extraction (UAE) [18,19,20,21,22], have been published. Such methods, especially UAE, are considered to be more productive and have attracted considerable interest in industrial applications [18,19,21,22,23,24,25]. UAE is considered one of the simplest, most efficient and most economical methods of increasing the yields of plant extraction [23]. UAE is also successfully employed in the extraction of the black locust flower [26] and wheatgrass [27] as well as the pot marigold flower [28] to investigate their polyphenols and flavonoids.

Ultrasound (US) effectively creates an acoustic cavitation effect that facilitates the penetration of the extraction solvent. This results in the intracellular content of the plant material being released successfully by increasing the solvent agitation, resulting in increased surface contact between the solvent and the target compounds, as well as increased solubility of the target compounds in the solvent [23]. Typically, UAE has a shorter extraction duration with a reduced usage of solvent compared to other traditional extraction methods, making it a green extraction technique that can be upscaled for industrial purposes [23]. Many methods have been developed to further selectively enrich a plant extract in bioactive compounds, including liquid-liquid extraction [15], solid phase extraction [29] or high-speed counter-current chromatography [30]. However, there are several limitations to these methods, such as low capacity, low yields and the need of special instrumentation [31]. Such methods also share common difficulties in completely extracting flavonoids, which are the main active ingredients in *N. lotus* stamens. By contrast, due to high adsorption efficiency, good stability, low operating costs and ease of use, macroporous resins (MPR) are effective for the enrichment of raw herbal extracts in bioactive components [31,32]. MPR has been successfully used in industries to separate and prepare bioactive extracts enriched in flavonoids, glycosides or saponins [31,32,33].

In this context, the present study reports on the establishment an UAE procedure, in conjunction with the MPR enrichment step, to increase the total flavonoid content (TFC) of *N. lotus* stamen extracts. First, design of experiment (DOE), coupled with the Response Surface Method (RSM), was used to determine and optimize the values of independent parameters of the UAE, including extraction duration, aqueous ethanol concentration (aqEtOH) and US frequency affecting the extraction of flavonoids from *N. lotus* stamens. The MPR purification step using microporous resin was also optimized with the evaluation of five different MPR. Analysis by LC-MS of this flavonoid-enriched extract from *N. lotus* stamens was performed. The in vitro cell-free and cellular antioxidant activities of each extract (UAE and UAE + MPR step) were evaluated, and then compared with activities obtained for extracts generated through conventional heat reflux extraction (HRE) and for commercial antioxidants.

## 2. Materials and Methods

### 2.1. Plant Materials and Plant Collection

The living specimen of *N. lotus* was searched and collected from its natural habitat in Nakhon Sawan province, Northern Floristic Regions of Thailand. The collected specimen was identified using the key-to-species and description in the existing Floras, as well as compared with the herbarium specimens kept in Forest Herbarium (BKF), the Pr. Kasin Suvatabandhu from Herbarium, Chulalongkorn University, (BCU). Herbarium abbreviations are used according to Thiers [34]. Then, the stamens from *N. lotus* flower were cut, and air-dried stamen samples were prepared following the World Health Organization [35] recommendations.

### 2.2. Chemicals

Analytical grade or the highest available purity solvents and reagents were used for extraction, LC-MS analysis and bioassays (Merck Millipore, Saint-Quentin Fallavier, France). Deionized water was prepared with the Milli-Q water purification system (Merck Millipore, Saint-Quentin Fallavier, France). Commercial standards were purchased from Extrasynthese (Genay, France).

### 2.3. Extraction

#### 2.3.1. Apparatus and General Procedure

The USC1200TH (Prolabo, Fontenay-sous-Bois, France) ultrasonic bath used is equipped with timer, frequency and temperature controllers, with a maximum heating power of 400 W corresponding to an acoustic power of 1 W/cm^2^ and a tank of 300 mm × 240 mm × 200 mm (inner dimension). The sample was placed in 5 mL quartz tubes containing 1 mL solvent for extraction and fitted with a vapor condenser. Their position in the ultrasonic bath was chosen in accordance with the Aluminum Foil Efficiency Test [18].

#### 2.3.2. UAE Optimization Using Full Factorial Design

Sample (100 mg) was suspended in 1 mL extraction solvent, corresponding to a liquid to solid ratio of 10:1 mL/g DW (dry weight), in 5 mL quartz tubes equipped with a vapor condenser. Extraction was done at 45 °C. Using XLSTAT2019 tools (Addinsoft, Paris, France), full factorial design experiment was applied to determine optimum extraction conditions. Three independent variables coded at three different levels (−1, 0 and +1; Table 1): aqueous ethanol (EtOH) concentration (X1), US frequency (X2) and extraction duration (X3) were considered. The experiments were randomized and carried out in triplicate. Equation fitting the model was calculated using XLSTAT2019 DOE Analysis tool (Addinsoft, Paris, France). Surface and contour plots showing TFC as a function of the coded levels of the independent variables were obtained using fxy Excel add-in (Redmond, WA, USA).

#### 2.3.3. Optimized UAE

Sample (100 mg) was suspended in 1 mL 90% (*v*/*v*) aqEtOH in 5 mL quartz tubes equipped with a vapor condenser. Extraction was performed at 45 °C in USC1200TH (Prolabo, Fontenay-sous-Bois, France) ultrasonic bath operating at a 30 kHz frequency for 46 min. After extraction, the extract was centrifuged 15 min at 5000× *g* (Heraeus Biofuge Stratos, Thermo Scientific, Illkirch, France) and the supernatant extract was filtered through 0.45 μm of nylon syringe membranes (Merck Millipore, Saint-Quentin Fallavier, France).

#### 2.3.4. Heat Reflux Extraction

Sample (100 mg) was suspended in 1 mL aqEtOH 90% (*v*/*v*) in 5 mL quartz tubes equipped with a vapor condenser. Extraction was performed at 45 °C in a water bath under agitation (150 rpm) for 46 min. After extraction, the extract was centrifuged 15 min at 5000× *g* (Heraeus Biofuge Stratos, Thermo Scientific, Illkirch, France) and the supernatant extract was filtered through 0.45 μm of nylon syringe membranes (Merck Millipore, Saint-Quentin Fallavier, France).

### 2.4. Determination of Total Flavonoid Content (TFC)

TFC was determined using the colorimetric aluminum trichloride (AlCl_3_) method [36]. Mixture (200 µL) was prepared in a microplate containing 20 µL of *N. lotus* stamen extract, 10 µL potassium acetate 1 M, 10 µL AlCl_3_ (10% (*w*/*v*)) and 160 µ of deionized water. Mixture was incubated 30 min at 25 °C in the dark, and an absorbance at 415 nm was determined with a microplate reader (Multiskan GO, Thermo Fischer Scientific, Illkirch, France). TFC was expressed in mg/g dry weight (DW) of quercetin equivalent using a five-point calibration line (linearity ranging from 0 to 40 µg/mL quercetin concentration with a R^2^ of 0.998).

### 2.5. LC-MS Analysis

Analysis of the LC-MS carried out, as described in Drouet et al. [37], on a Water 2695 Alliance coupled with a single quadrupole mass spectrometer ZQ (Waters-Micromass, Manchester, UK). LC-ESI-MS (liquid chromatography-electrospray ionization-mass spectrometry) MassLynx 4.0 software (Waters-Micromass, Manchester, UK) was used to acquire and process data. The separation was conducted using a linear gradient: from a mixture of 10:90 (*v*/*v*) to 100:0 (*v*/*v*) of solvent A (methanol) and solvent B (water + 0.05% (*v*/*v*) formic acid) at a flow rate of 1.00 mL/min during a run of 1 h. The detection was set at 350 nm. Before injection, extracts were centrifuged 15 min at 5000× *g* (Heraeus Biofuge Stratos, Thermo Scientific, Illkirch, France) and the supernatant extract was filtered through 0.45 μm of nylon syringe membranes (Merck Millipore, Saint-Quentin Fallavier, France).

### 2.6. Macroporous Resin (MPR) Purification Step

#### 2.6.1. MPR Preparation

Five MPR purchased from (Merck Millipore, Saint-Quentin Fallavier, France) presented in Table 2 were evaluated for flavonoid purification step. Before use, the MPR was activated by soaking for 24 h with 95% (*v*/*v*) aqEtOH and washed thoroughly with deionized water afterwards. The MPR was then subsequently soaked 12 h in 5% HCl and then 2% NaOH solutions. Afterward, MPR was extensively washed with deionized water until neutral.

#### 2.6.2. MPR Selection

The adsorption and desorption ratios were evaluated and compared among the five MPRs for selection. In this screening phase, absorption and desorption were performed at 25 °C. Each prepared MPR (5 g) was placed in an Erlenmeyer flask and mixed with 50 mL of *N. lotus* stamen extract prepared using optimized UAE conditions. At 25 °C in the dark, each flask was then incubated on an orbital shaker operating at 120 rpm. For the screening phase, incubation time to achieve absorption equilibrium was arbitrarily fixed to 6 h. Through filtration under vacuum, the resultant liquid phase was isolated from the MPR and its TFC was determined. The absorption ratio A was calculated as follow: A (%) = [(C_0_ − C_1_)/C_0_] × 100, where C_0_ (expressed in mg/mL) is the initial total flavonoid concentration of the extract and C_1_ (expressed in mg/mL) is the equilibrium total flavonoid concentration.

The MPR was washed with deionized water to eliminate excess of flavonoids. Then, it was desorbed with 50 mL of 95% (*v*/*v*) aqEtOH solution with incubation at 25 °C in an orbital shaker operating at 120 rpm. For the screening phase incubation time to achieve desorption was arbitrary fixed to 6 h. The TFC of the resulting eluent was determined. The desorption ratio D was calculated as follow: D (%) = [(V_2_ × C_2_)/(M × Q)] × 100, where C_2_ (expressed in mg/mL) is the total flavonoid concentration of the eluent, V_2_ (in mL) is the volume of the eluent solution, M (in g) is the MPR mass and Q is the absorption capacity determined as follow: Q = [(C_0_ − C_1_)/M] × V_1_, with V_1_ (in mL) is the initial volume of the extract placed in contact with the MPR.

#### 2.6.3. Optimization of Static Absorption and Desorption on XAD-8 MPR

After the screening phase, the XAD-8 MPR was selected for further optimization experiments. Optimal absorption and desorption times were determined as described above with different incubation durations (0–60 min) for absorption and then for desorption. The concentration of the aqEtOH solution (0–100 (*v*/*v*)) as well as the desorption incubation temperature (25–55 °C) were also evaluation using the optimal absorption incubation time of 25 min and the optimal desorption incubation time of 15 min. Using 1 M HCl and 1 M NaOH solutions, the desorption solution pH was adapted to different values (2–10) to evaluate the influence of the pH value on purification capacity of XAD-8 MPR.

Estimation of non-target compounds, such as reducing sugars, was done by the determination of conductivity values as well as total reducing sugar content as described previously [38,39].

### 2.7. Antioxidant Assays

#### 2.7.1. In Vitro Cell Free DPPH Free Radical Scavenging Assay

The DPPH (2,2-diphenyl-1-picrylhydrazyl) in vitro cell free cell assay was used to evaluate the free radical scavenging activity of the extracts as described in Shah et al. [40]. In brief, 20 μL of extract were mixed with 180 μL of DPPH reagent solution (0.1 mM final concentration in methanol) in a microplate well, and incubated for 60 min in the dark at 25 °C. Butylated hydroxytoluene (BHT, 100 µM in methanol) was used as positive control. Negative control was obtained with 180 µL DPPH mixed with 20 μL of the corresponding extraction solvent. After incubation, absorbance at 515 nm was recorded with a microplate reader (BioTek ELX800 Absorbance Microplate Reader, BioTek Instruments, Colmar, France). Antioxidant capacity was expressed as Trolox C equivalent antioxidant capacity (TEAC) with a standard curve (0–500 μM Trolox C; R^2^ = 0.999).

#### 2.7.2. In Vitro Cell Free ABTS Antioxidant Assay

The ABTS (2,2-azinobis (3-ethylbenzthiazoline-6-sulphonic acid)) in vitro cell free cell assay was used to evaluate the free radical scavenging activity of the extracts as described in Ullah et al. [41]. First, the absorbance at 734 nm of the ABTS solution (ABTS salt (7 mM) and potassium persulphate (2.45 mM), incubated in the dark for at least 16 h) was adjusted to 0.7 after its preparation. Then, 10 μL of extract were mixed with 190 µL of this prepared ABTS solution in a microplate well, and incubated for 15 min in the dark at 25 °C. Butylated hydroxytoluene (BHT, 100 µM in methanol) was used as positive control. Negative control was obtained with 190 µL ABTS solution incubated with 10 μL of the corresponding extraction solvent. After incubation, absorbance at 734 nm was recorded with a microplate reader (BioTek ELX800 Absorbance Microplate Reader, BioTek Instruments, Colmar, France). Antioxidant capacity was expressed as Trolox C equivalent antioxidant capacity (TEAC) with a standard curve (0–500 μM Trolox C; R^2^ = 0.998).

#### 2.7.3. In Vitro Cell Free FRAP Antioxidant Assay

The FRAP (Ferric Reducing Antioxidant Power) in vitro cell free cell assay was used to evaluate the free radical scavenging activity of the extracts as described in Abbasi et al. [42]. In brief, 10 μL of extract were mixed well with FRAP solution (FeCl_3_ (20 mM), 2,4,6-Tris(2-pyridyl)-*s*-triazine (TPTZ, 10 mM), acetate buffer (300 mM, pH 3.6) prepared in a 1:1:10 (*v*/*v*/*v*) ratio) in a microplate well, and incubated for 15 min in the dark at 25 °C. Butylated hydroxytoluene (BHT, 100 µM in methanol) was used as positive control. Negative control was obtained with 190 µL FRAP solution incubated with 10 μL of the corresponding extraction solvent. After incubation, absorbance at 630 nm was recorded with a microplate reader (BioTek ELX800 Absorbance Microplate Reader, BioTek Instruments, Colmar, France). Antioxidant capacity was expressed as Trolox C equivalent antioxidant capacity (TEAC) with a standard curve (0–500 μM Trolox C; R^2^ = 0.998).

### 2.8. Cellular Antioxidant Assay

For evaluation of cellular antioxidant activity, an assay based on the method defined by Nazir et al. [43], using yeast cells, was employed. Yeast cells (DBY746 (*MATα* *leu*2-3,112 *his*3Δ1 *trp*1-289a *ura*3-52 GAI+)) were grown aerobically in complete 2.0% (*w*/*v*) glucose YPD (yeast extract peptone dextrose) medium (Sigma Aldrich, Saint-Quentin Fallavier, France) in an orbital shaker (150 rpm) at 30 °C. Each extract was evaporated under nitrogen flow, dissolved in DMSO, and then added to the yeast cells 6 h prior oxidative stress induction at a final concentration of 10, 25 or 50 µg/mL. The same volume of DMSO was used for untreated control yeast cells, whereas yeast cells treated with resveratrol (Sigma Aldrich, Saint Quentin Fallavier, France) at 10 μM, with the final concentration prepared in DMSO, was used as positive control. The final concentration of DMSO applied on the yeast cells was circa 1% (*v*/*v*). Oxidative stress was induced by UV-C irradiation at 106.5 J/m^2^ UV-C (254 nm) using Vilber VL-6.C filtered lamp (Thermo Fisher Scientific, Villebon-sur-Yvette, France). Yeast cells were then incubated overnight at 30 °C.

Reactive oxygen and nitrogen species (ROS/RNS) produced were determined using dihydrorhodamine-123 (DHR-123) fluorescent dye (Sigma-Aldrich, Saint-Quentin Fallavier, France) as described by Tungmunnithum et al. [25]. Approximately 10^8^ yeast cells of each condition were rinsed twice with phosphate buffered saline (PBS 1X, pH7.4), before being resuspended in 0.4 μM DHR-123 solution prepared in PBS (1X, pH7.4) and then incubated 10 min at 30 °C in the dark. After twice washing with PBS (1X, pH7.4), the fluorescence intensity was measured with a VersaFluor fluorimeter (Biorad, Marnes-la-Coquette, France) using λex = 505 nm and λem = 535 nm.

A thiobarbituric acid reactive substances (TBARS) assay was employed to determine the membrane lipid peroxidation level. For this purpose, ca. 10^7^ cells were ground in liquid nitrogen with a mortar and pestle, dissolved in 250 µL double distilled water (molecular biology grade, Thermo Fisher Scientific, Villebon-sur-Yvette, France), and centrifuged for 10 min at 10,000× *g*. Fractions of the supernatant (75 µL) were then mixed with 25 µL of SDS (3% (*w*/*v*)), 50 µL of TBA (thiobarbituric acid, 3% (*w*/*v*) in a 50 mM NaOH solution), and 50 µL of HCl (23% (*v*/*v*)) with vigorous mixing after each addition. The mixture was incubated at 80 °C for 20 min and then cooled on ice. The TBARS value was determined by measuring absorbance at 532 nm, and subtracting non-specific absorbance at 600 nm using UV-Vis spectrophotometer (Cary50, Varian, Les Ulis, France).

Impact on gene expression was determined by RTqPCR. First, total RNAs were extracted from the yeast cells with the RiboPure RNA extraction kit (Thermo Scientific, Illkirch, France) following manufacturer instructions. Total RNA content was determined with the Quant-iT HR RNA assay and using Qubit fluorimeter (Thermo Scientific, Illkirch, France). Then, reverse transcription was completed with the SuperScript IV cDNA synthesis kit (Thermo Scientific, Illkirch, France) using 5 mg of total RNA, oligo (dT) adaptor primer (Thermo Scientific, Illkirch, France) and 1 unit of RiboLock (Thermo Scientific, Illkirch, France). Real-time PCR was performed with a PikoReal™ Real-Time PCR System (Thermo Scientific, Illkirch, France) using DyNAmo ColorFlash SYBR Green qPCR (Thermo Scientific, Illkirch, France) and specific primers (Eurogentec, Liege, Belgium). Primers used were: *SIR2*, forward: 5′-CGTTCCCCAAGTCCTGATTA-3′, and reverse: 5′- CCACATTTTTGGGCTACCAT-3′; *SOD2*, forward: 5′-CTCCGGTCAAATCAACGAAT-3′, and reverse: 5′-CCTTGGCCAGAAGATCTGAG-3′; *TUB1*, forward: 5′-CCAAGGGCTATTTACGTGGA-3′, and reverse: 5′-GGTGTAATGGCCTCTTGCAT-3′. The parameters used for the qPCR were as follows: 95 °C—5 min initial denaturation, then 40 cycles of 95 °C—15 s denaturation, 55 °C—10 s primer annealing and 72 °C—20 s extension. After these 40 cycles, a final extension period 72 °C—90 s was carried out. The presence of a single amplicon was confirmed by observation of a single peak in the melting curve obtained after amplification for each gene and condition. Housekeeping gene *TUB1* was used for normalization. Expression levels were calculated and normalized using 2^−ΔΔCt^ method.

### 2.9. Statistical Treatment of Data

Statistical analyses were performed with XLSTAT 2019 suite (Addinsoft, Paris, France). Data composed of at least three independent replicates were presented using the means and standard deviations. Student *t*-test was carried out for statistical comparative analysis. Significant thresholds at *p* < 0.05, 0.01 and 0.001 were represented by *, ** and ***, respectively. Different letters were used to indicate significant thresholds at *p* < 0.05.

## 3. Results and Discussion

### 3.1. Optimization of UAE of Total Flavonoids from N. lotus Stamens

In view of its high reproducibility due to the actual measurement of a large number of experimental conditions compared to other DOE approaches [44], a full factorial design was used to optimize the UAE of *N. lotus* stamens flavonoids. In the framework of the development of the UAE method, three parameters of influence stand out very clearly: the nature of the extraction solvent, the US frequency and the extraction duration [18,21,22,23,24,25]. With these considerations in mind, the impact of these three variables for the development of an UAE of the total flavonoids from *N. lotus* stamen was evaluated. When designing an extraction method, the choice of solvent is a key parameter to determine. Ethanol has been widely used as environmentally friendly solvents for extracting a wide variety of polyphenols from plant matrices [17,18,21,22,25,45,46,47]. EtOH is one of the least toxic to humans and more environmentally friendly organic solvents [45], classified by the Food and Drug Administration (FDA) as a generally recognized as safe (GRAS) substance [48]. It is a versatile solvent with both a polarity and extraction capacity that can be easily modulated by the simple addition of water, making it the ideal solvent for extracting a wide variety of low-to high-polarity polyphenols. Water and EtOH are commonly used as two universal solvents for various food and/or cosmetic applications [21,22,23,45]. It was therefore rational for us to choose these two universal solvents for the development of the UAE method of total *N. lotus* stamen flavonoids according to the principles of green chemistry.

The three variables were: X1 for aqEtOH concentration (ranging from 50 to 100% (*v*/*v*), X2 for US frequency (ranging from 0 (no US applied) to 45 kHz) and X3 extraction duration (ranging from 20 to 60 min). Their coded levels and experimental values are shown in Table 1. The ranges of each variables were determined based on single parameter evaluation preliminary experiments. In comparison, based on the results obtained from these preliminary experiments, the influences of the liquid/solid ratio and extraction temperature parameters were negligible (i.e., no significant impact) and were fixed to 25:1 mL/g DW and 45 °C, respectively (data not shown). In silico, the 27 different bath conditions (run ID) for the full factorial design were determined and randomized (run order). The respective independent process variables for each batch condition are shown in Table 2. Each batch condition was tested in separate triplicates. Table 3 presents the TFC from the *N. lotus* stamens resulting from these extraction conditions. Here, TFC ranged from 29.68 ± 2.75 (run ID#1) to 230.74 ± 5.97 (run ID#27) mg/g DW. These results clearly have shown that stamens are a rich flavonoid plant tissue. This is quite logical given the key biological role of stamens (bearing pollen) in which flavonoids may have important plant functions as antioxidants (e.g., during pollen germination [49]) but also as protective compounds (e.g., antifungal [50]), particularly for aquatic plants such as water lily.

The multiple regression analysis (Table 4) provided a model of the TFC extracted from *N. lotus* stamens as a function of the three different variables. The TFC extraction yield (Y_TFC_), obtained using the conditions listed in Table 1; Table 2, was expressed in the form of a polynomial equation, Y_TFC_ as a function of the X1 (aqEtOH concentration), X2 (US frequency) and X3 (extraction duration): Y_TFC_ = 187.5 + 78.0 X_1_ + 16.5 X_2_ + 3.0 X_3_ – 42.4 X_1_^2^ – 22.7 X_2_^2^ – 6.7 X_3_^2^ + 15.7 X_1×2_ + 3.6 X_1_X_3_ – 3.8 X_2_X_3_ (Table 4).

The statistical analysis (Table 4) evidenced the highly significant important effect (*p* < 0.001) on TFC extracted from *N. lotus* stamens of aqEtOH concentration and US frequency through their linear coefficients X_1_ and X_2_, quadratic coefficients X_1_^2^ and X_2_^2^, as well as their interaction coefficient X_1_X_2_. On the contrary, all the other coefficients involving extraction duration (i.e., X_3_) were not statistically significant (*p* > 0.05). The representation normalized coefficients for each variable, with the 95% confidence interval (CI), for the proposed polynomial model is shown in Figure S2.

Table 5 lists the results of the analysis of variance (ANOVA) and the fit for the model obtained for TFC extracted from *N. lotus* stamens. This analysis, in particular the high *F*-value (239.686) and the low *p*-value (*p* < 0.0001), clearly indicated that the model as highly significant and appropriate to predict the TFC extracted from the *N. lotus* stamens as a function three variable value with high precision (Table 4). This trend is also confirmed by the low and non-significant lack of fit value, and the coefficient of determination of the model (R^2^ of 0.992 with an adjusted value at 0.988), whereas the coefficient value (CV) indicated the appropriateness between the model and the experimental values. This precision of the model in the prediction of TFC experimental values is further illustrated by the predicted vs. experimental TFC plot presented in Figure S1.

The values of linear coefficients of the polynomial second-order equation for X_1_ aqEtOH concentration, X_2_ US frequency and X_3_ extraction duration, as well as of the interaction coefficients X_1_X_2_ (aqEtOH concentration × US frequency) and X_1_X_3_ (aqEtOH concentration × extraction duration), were all positive, suggesting that the increase of these parameters results in a favorable effect on the extraction of TFC. However, the negative values of their quadratic coefficients (X_1_^2^, X_2_^2^ and X_3_^2^, respectively), as well as of the interaction coefficient between US frequency and extraction duration (X_2_X_3_), imply that this total flavonoids extraction process from the stamens of *N. lotus* is much more complex and reaches a maximum value before decreasing for high values of those three extraction parameters.

**Figure 2:**
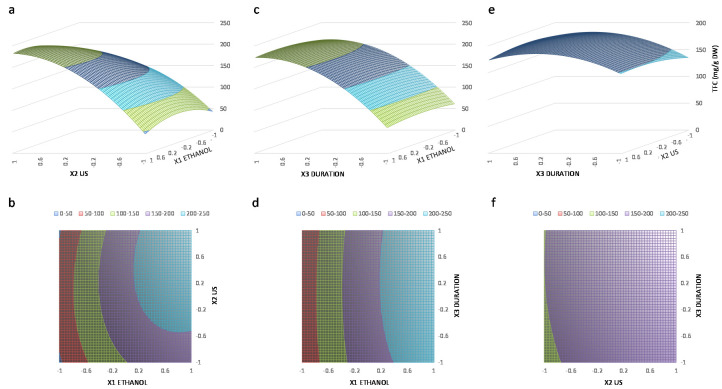
Three-dimensional (3D) surface response and two-dimensional (2D) contour plots, respectively, from the model predicted TFC (in mg/g DW) extracted from mature *N. lotus* stamens as a function of aqEtOH concentration and US frequency. (**a**,**b**), aqEtOH concentration and extraction duration (**c**,**d**) and US frequency and extraction duration (**e**,**f**).

These trends are clearly observed in the three-dimensional (3D) plots (Figure 2), with first positive action on the TFC extracted from *N. lotus* stamens with increased aqEtOH concentrations combined with higher US frequency and/or longer duration of extraction prior to its decline (Figure 2a,b). High US frequency combined with prolonged extraction duration at high US frequency, resulted in a marked decline in TFC extracted from *N. lotus* stamens (Figure 2c).

EtOH is a universal solvent that is widely used to extract a wide range of phenolic compounds of low to high polarity, since its polarity and thus its extraction capacity can be easily modulated by adding water. Results indicate that a small addition of water, and therefore a slight increase in polarity, is favorable for the extraction of total flavonoid from *N. lotus* stamens. In accordance with this result, different organs of *N. lotus* have been reported to accumulate flavonoids mainly in the form of glycosides [4,14,15]. Solvents used for flavonoid extraction are generally selected according to their polarity [51]. The less polar solvents are particularly useful for the extraction of aglycones, while more polar solvents are used if glycosides are pursued [51]. As is the case in the present study, flavonoid glycosides are typically isolated from plant material by extraction with alcohol, such as EtOH, water or a combination thereof [17,18,21,22,25,45,46,51]. Note that the concentration of aqEtOH depends also on the plant matrix considered for optimum results [23,45]. The cavitation effect and the diffusion coefficient of the compounds in the extraction solvent are significantly affected by the US frequency [23,45]. As a result, the US frequency may increase the solubilization of the compound in the extracting solvent and thus improve the extraction yield [23,45]. By acting on the cavitation effect and the diffusion coefficient, the US frequency also contributes to the reduction of the extraction duration [23,45]. The duration of extraction, in itself, is also an important parameter to consider, bearing in mind that a duration increase does not automatically increase the extraction yield, particularly during UAE. In fact, extended extraction duration in the case of UAE may lead to increased degradation of the bioactive compounds [21,25]. Extended extraction duration during UAE, particularly in the presence of water has been shown to induce oxidation of polyphenols, thus drastically reducing antioxidant capacity of the resulting extract [21,23]. In the context of green chemistry, it is also of particular interest to reduce the extraction duration in order to diminish the impact of energy consumption [52]. Consequently, all these extraction parameters should be precisely optimized and their possible interactions taken into account in order to avoid any sharp reduction in the extraction yield (both quantitatively and qualitatively) but also any dramatic decline in the biological activity of the sample extract.

Hence, in this context, the use of multivariate techniques, such as full factorial design, to optimize the UAE method starting from complex plant materials is particularly appropriate [44,53]. It is an effective, precise and rapid way of integrating a large number of extraction conditions and of demonstrating possible interactions between independent variables compared to single-factor approaches [44,53]. Here, according to the adjusted second order polynomial equation determined by the TFC obtained with the full factorial design experiment, the optimum conditions were: 90% (*v*/*v*) aqEtOH as extraction solvent, 34.6 kHz for the US frequency and an extraction duration of 46 min (here, using an extraction temperature and liquid to solid ratio fixed at 45 °C and 25:1 mL/g DW, respectively). Adjusted to the US bath apparatus used, an US frequency of 30 kHz was used. Under these optimized conditions, TFC extracted from *N. lotus* stamens reached 235.45 ± 5.44 mg/g DW.

### 3.2. Optimization of Macroporous Resin Purification of Total Flavonoids from N. lotus Stamens

**Figure 3:**
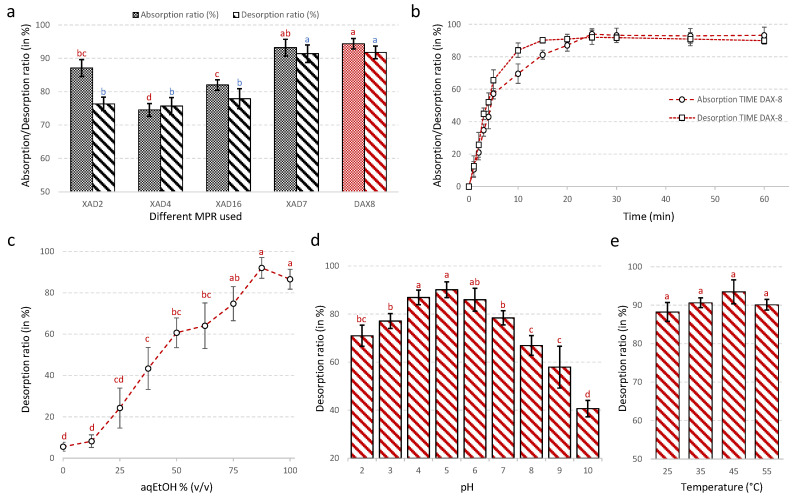
Static adsorption and desorption capacities of the five tested microporous resins for flavonoids from *N. lotus* stamens. (**a**); Kinetics for static adsorption and desorption on DAX-8 resins for flavonoids from *N. lotus* stamens (**b**); Effect of ethanol concentration on desorption ratio on DAX-8 resins for flavonoids from *N. lotus* stamens (**c**); Effect of pH on desorption ratio on DAX-8 resins for flavonoids from *N. lotus* stamens (**d**); Effect of temperature on desorption ratio on DAX-8 resins for flavonoids from *N. lotus* stamens (**e**).

Five MPR (Table 2) with different surface areas, average pore diameters, matrix types and polarities were investigated for the purification of total flavonoids from *N. lotus* stamen. The results of their adsorption capacity and desorption capacity of the total flavonoids from *N. lotus* stamen extract obtained under optimal UAE conditions are shown in Figure 3a. Both DAX-8 and XAD-7 acrylic-type MPR showed stronger static adsorption and desorption for the flavonoids from *N. lotus* stamen extract than other styrene divinyl benzene resins tested. The styrene-divinyl-benzene macroreticulate XAD-2 MPR showed similar absorption capacity compared to the XAD-7, but with significantly lower desorption capacity than these two microporous resins. Due to its higher adsorption and desorption capacity for the flavonoids from *N. lotus* stamen extract, the DAX-8 MPR was selected. The purification conditions were further optimized to make the purification process more efficient.

Figure 3b shows the kinetics for static adsorption and desorption capacity of the DAX-8 MPR for total flavonoid present in *N. lotus* extract from stamens. As shown by the adsorption curve, total flavonoids from the *N. lotus* extract were rapidly and efficiently absorbed by the DAX-8 MPR with an adsorption equilibrium occurring after 25 min, and a maximal adsorption capacity of 94.37 ± 3.30% for the total flavonoids from the extract. The desorption ratio increased sharply, reaching a maximum value after 15 min (Figure 3b). The desorption curve indicates that the total flavonoids absorbed into the resin were more effectively desorbed with 90% (*v*/*v*) aqEtOH solution (Figure 3c). The pH of the extract significantly influenced this purification step with optimal value obtained pH5 (Figure 3d). Increased desorption temperature to 45 °C resulted in a slight but not significant increase in the desorption ratio (Figure 3e). Under these conditions, the desorption ratio reached 94.37 ± 1.56%.

Divergent assumptions on the effect of the chemical structure (e.g., styrene, acrylic) and physical properties (such as surface area, pore diameter) of the MPR on the recovery of flavonoids were made, depending on the plant matrix [54,55,56,57,58,59,60]. In line with some recent reports [56,58,59,60], here, better results were achieved using the moderately polar acrylic DAX-8 and XAD-7 MPRs, certainly due to the nature of the flavonoids present in the *N. lotus* stamen extract. Acrylic MPRs, DAX-8 MPR in particular, have previously been reported for its efficacy in the enrichment of total flavonoid extracts from mature oil palm leaf (*Elaeis guineensis* Jacq.) [60]. The authors of this study pointed out the importance of adjusting the chemical nature of the MPR, but also its physical properties, to the nature of the plant extract and the compounds to be purified [60]. The ester substitution of the acrylic matrix with a lower surface area (140 m^2^/g), a lower particle diameter (250–420 μm) and a lower pore diameter (225 Å) of the DAX-8 MPR compared to the XAD-7 MPR were more suitable characteristics for the purification of total flavonoids from *N. lotus* stamen extract. The rapid equilibrium reached for both absorption (i.e., 25 min) and desorption (i.e., 15 min) are in line with the literature data [55]. The results showed that concentration and pH of the aqEtOH solution and the temperature influenced the desorption capacity of the MPR. In the literature, most flavonoids are desorbed with an aqEtOH concentration for the desorption solution ranging from 75–100% (*v*/*v*) in agreement with the present study [54,55,56,57,58,59,60]. The pH value can change the ionization of flavonoids, which also affects their adsorption to the MPR [57]. The optimal pH value was around 5, which resulted in an increased enrichment of the *N. lotus* stamen extract in total flavonoids. This result is consistent with other studies [55,56,57,58,59,60]. At a higher pH value, the flavonoid phenolic hydroxyl groups dissociated with H+ and the corresponding flavonoid anion result in lower adsorption capacity and thus a drastic reduction in the purification yield [57].

### 3.3. Comparison with Conventional HRE Method

**Figure 4:**
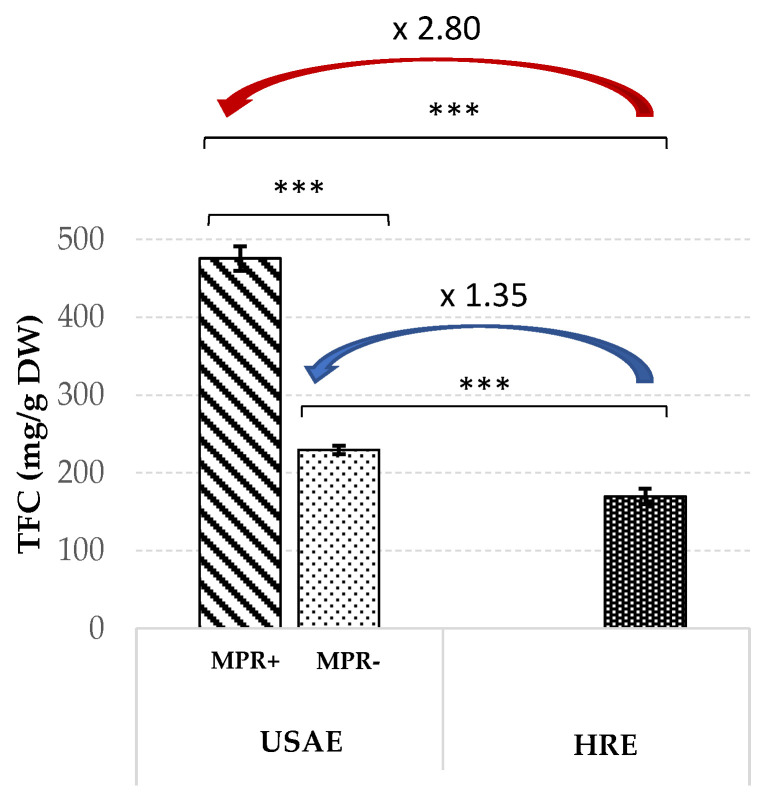
Comparison of TFC extracted from *N. lotus* stamens using traditional HRE and presently optimized UAE alone (MPR−) or coupled with the optimized DAX-8 macroporous resin purification step (MPR+). Values are means ± SD of three independent experiments. *** significant *p* < 0.001.

To evaluate the efficacy of the proposed protocol, TFC obtained from *N. lotus* stamens using optimized UAE alone (UAE MPR−) or coupled with the proposed MPR purification step (UAE MPR+) was compared to the conventional HRE method. For this purpose, HRE was performed using the same aqEtOH concentration of 90% (*v*/*v*), extraction duration of 46 min, temperature of 45 °C and L/S ratio of 25:1 mL/g as for the optimized UAE (the only difference is the absence of a request from the US). The results of these extraction protocols are shown in Figure 4.

The results of these different extraction procedures showed a significant increase (1.35-fold) in TFC extracted from *N. lotus* stamen using UAE (TFC = 235.45 ± 5.44 mg/g DW) compared to HRE (TFC = 169.64 ± 9.86 mg/g DW), thus demonstrating the efficiency of US application. Higher extraction yields can be obtained with HRE when the extraction duration increases, but without reaching observed values with the UAE (data not shown), and with increased energy consumption as a result of this extension in duration. Therefore, in the context of green chemistry, but also for potential industrial applications, this UAE protocol is of particular interest in terms of reducing energy consumption through the use of this innovative technology. It enables high extraction yields of flavonoids from *N. lotus* stamens with lower extraction costs (reduction in terms of length of treatment and solvent use). It can be assumed that this UAE efficiency may be a consequence of the collapse of cavitation bubbles acting as microreactors creating locally high temperature and pressure conditions in the surrounding liquid [23], resulting in a more effective breakdown of the plant tissue and thus a more efficient release and solubilization of the released flavonoids. This increase yield appeared even higher with UAE coupled with MPR purification (TFC = 475.42 ± 16.61 mg/g DW), resulting in a significant 2.80-fold increase compared to HRE. This enrichment in flavonoids was the result of the UAE, followed by the MPR purification step. The enrichment in total flavonoids of the plant extract obtained by the UAE following the MPR purification step has already been reported [31]. Here, this enrichment may result from the elimination of other non-target compounds from the extract, such as simple sugars, as demonstrated by the reductions observed in the conductivity value as well as total sugar content of the extract following the XAD-8 MPR purification step (Table S1).

### 3.4. Analysis of Flavonoids by LC-MS

**Figure 5:**
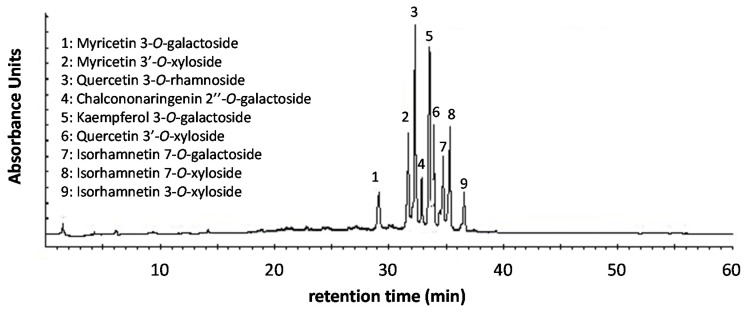
Representative LC chromatogram of extract from *N. lotus* stamens prepared by UAE followed by MPR purification step recorded at 350 nm.

Figure 5 shows the chromatographic LC profile recorded at 350 nm of an extract from *N. lotus* stamens prepared, under optimum conditions, by UAE followed by DAX-8 MPR purification step.

Based on LC-MS analysis, a comparison with authentic standards and literature data [4,14], nine major flavonoids derived from *N. lotus* extracts were identified in the MPR extract (Table S2). These flavonoids were: (i) eight flavonol glycosides: three isorhamnetin (Iso) derivatives (Iso-7-O-galactoside (7), Iso-7-O-xyloside (8) and Iso-3-O-xyloside (9)), two myricetin (Myr) derivatives (Myr 3-O-galactoside (1) and Myr 3′-O-xyloside (2)), two quercetin (Que) derivatives (Que-3-O-rhamnoside (3) and Que-3′-O-xyloside (6)) and one kaempferol derivative (Kae-3-O-galactoside (5)); (ii) one chalcone glycoside: chalcononaringenin-2′′-O-galactoside (4) (Figure 5). Their relative quantification in the different *N. lotus* extracts is presented in Table 6. Previously, the current LC separation conditions were optimized for the separation of different plant extracts, in particular for the separation of simple phenolic glycosides that elute between 0 and 25 min, the flavonoid glycosides between 25 and 45 min, while the flavonoid aglycones are separated after 45 min (unpublished results). Here, the current separation clearly showed that the UAE and DAX-8 MPR purification steps are effective procedures for enriching *N. lotus* extract from stamen in flavonoid glycosides. All these flavonoids have been previously described in *N. lotus*, especially in flower tissue [4,14], but the current study is the first one specifically dedicated to the extraction from stamens of antioxidant flavonoids. The stamen is the reproductive organ of a flower that produces pollen. In plants, flavonols accumulated in pollen grains are known to enhance their development and regulate the sexual reproduction of plants by reducing the abundance of reactive oxygen species (ROS) [49]. Bees also collect this pollen from plant stamens (in the anthers) to make a product rich in antioxidant flavonoids consumed by humans with many human health-related properties [50].

### 3.5. Evaluation of Antioxidant Activity of Total Flavonoid Extracts from N. lotus Stamens

#### 3.5.1. In Vitro Antioxidant Activity of Total Flavonoid Extracts from *N. lotus* Stamens

The antioxidant activity of plant extracts cannot be assessed by a single method due to the complex nature of the phytochemicals, in particular because the determination of antioxidant activity is highly dependent on the reaction mechanism involved. For this reason, several chemical or biological assays are required to assess antioxidant activity and describe the antioxidant mechanism of action of a plant extract [61]. In vitro cell-free chemical assays based on different mechanisms of reaction may provide an idea of the chemistry behind a plant extract’s antioxidant activity. These in vitro cell-free antioxidant assays can be roughly divided into different categories based on the chemical reaction involved, with ABTS based on a hydrogen atom transfer reaction (HAT) and FRAP based on an electron transfer reaction (ET), while DPPH can be considered as a mixed assay [61,62,63]. With this in mind, a series of in vitro cell-free antioxidant assays, including DPPH and ABTS scavenging activities, and a FRAP assay were used to determine the antioxidant activity of the extracts from *N. lotus* stamens. Results, shown in Table 7, were expressed as Trolox C equivalent antioxidant activity (TEAC), with the synthetic commercial antioxidant BHT being used as positive control.

All *N. lotus* extracts showed good free radical scavenging activity in the DPPH assay. The activity of *N. lotus* extract resulting from UAE followed by DAX-8 MPR purification step (MPR condition: 4749.67 ± 166.05 μmol TEAC/g, Table 6) was significantly higher than that of the positive control BHT (3059.07 ± 401.32 μmol TEAC/g). The free radical scavenging activity in the DPPH assay of *N. lotus* extract resulting from UAE without additional purification step (UAE condition: 3167.85 ± 342.64 μmol TEAC/g, Table 6) was similar to that of BHT. A similar trend was observed for the FRAP assay, in particular, with *N. lotus* extract resulting from UAE followed by DAX-8 MPR purification step (MPR condition: 3601.74 ± 126.26 μmol TEAC/g) showing significantly higher activity than that of BHT (1724.12 ± 161.27 μmol TEAC/g). Using ABTS assay, the antioxidant activity recorded for *N. lotus* extract resulting from UAE followed by DAX-8 MPR purification step (MPR condition: 4710.67 ± 155.64 TEAC/g) was statistically similar to that of BHT (4378.12 ± 106.62 μmol TEAC/g). These results highlighted the interest of the proposed extraction/purification process with a great antioxidant potential, at least similar to that of BHT. From a mechanistic point of view, this antioxidant activity can be correlated with the various flavonoid components found in this *N. lotus* extract, in particular with the capacity of electron donation, which have been associated with the degree and position of hydroxylation and methoxylation of the flavonoid ring B [64].

#### 3.5.2. Cellular Antioxidant Activity of Total Flavonoid Extracts from *N. lotus* Stamens

Although interesting from a purely predictive point of view based on chemical reactions, the in vitro cell-free assays do not necessarily represent the situation occurring in in vivo systems. Therefore, the validity of these antioxidant data must be considered to be limited to an interpretation within the significance of the chemical reactivity in relation to the considered radicals generated in vitro, and therefore, this must be confirmed in vivo. Consequently, the antioxidant activity of the three *N. lotus* extracts was further studied for their ability to inhibit ROS/RNS production as well as membrane lipid peroxidation in a cellular oxidative stress model, to have an improved understanding of and better reflect the in vivo situation.

**Figure 6:**
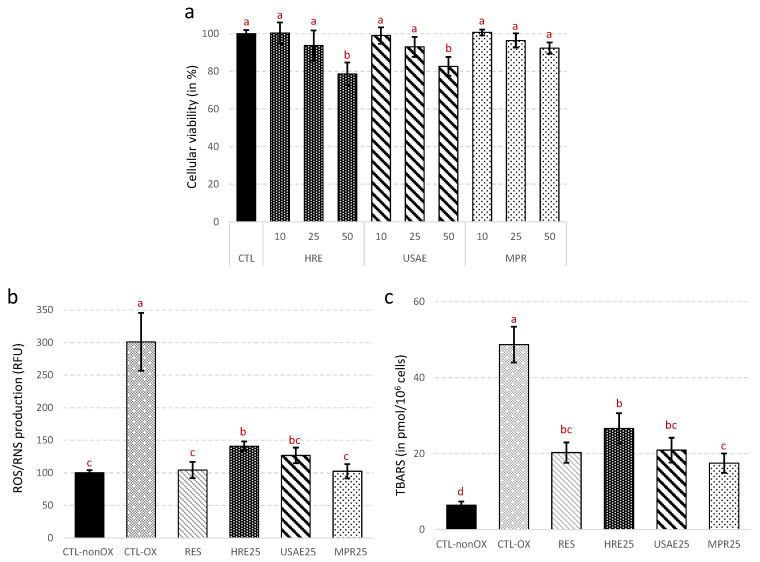
Effects of the different extracts from *N. lotus* stamen on yeast cell viability estimated at different extract concentrations (10, 25 or 50 µg/mL) (**a**), ROS/RNS production in response to UV-induced oxidative stress (**b**) and TBARS production in response to UV-induced oxidative stress (**c**). CTL-nonOX are control yeast cells; CTL-OX are control yeast cells subjected to UV-induced oxidative stress; RES are yeast cells subjected to UV-induced oxidative stress in presence of resveratrol (positive antioxidant control, 10 µM); HRE25 are yeast cells subjected to UV-induced oxidative stress in presence of 25 µg/mL of *N. lotus* extract obtained by HRE; UAE are yeast cells subjected to UV-induced oxidative stress in presence of 25 µg/mL of *N. lotus* extract obtained by UAE; MPR are yeast cells subjected to UV-induced oxidative stress in presence of 25 µg/mL of *N. lotus* extract obtained by UAE followed by DAX-8 MPR purification step. Values are means ± SD of three independent experiments. Different letters represent significant differences between the various extraction conditions (*p* < 0.05).

In order to avoid any bias resulting from possible toxic or antifungal activities, the absence of any significant effects on the growth and viability of the extracts was evaluated prior to the assessment of their cellular antioxidant action. The absence of a toxic effect was recorded, for the three extract concentrations tested, for the *N. lotus* extract resulting from UAE followed by DAX-8 MPR purification step (MPR, Figure 6a). On the contrary, a slight toxic effect was observed with *N. lotus* extracts resulting from both UAE (without DAX-8 MPR purification step, UAE, Figure 6a) and HRE at the highest concentration evaluated (i.e., 50 µg/mL). However, for these extracts, no significant impact on cell viability were observed for the other two concentrations. A concentration of 25 µg/mL was therefore used to evaluate cellular antioxidant activity of each extract.

The production of ROS and RNS in yeast cell subjected to oxidative stress induced by UV treatment was assessed using a dihydrorhodamine 123 (DHR123) probe (Figure 6b). In response to the UV treatment, ROS and RNS production increased in control cells. As for resveratrol (RES, positive control, Figure 6b), the three different *N. lotus* extracts were able to significantly reduce the production of ROS and RNS in yeast in response to UV treatment. *N. lotus* extracts from UAE (both resulting from UAE and MPR, Figure 6b) inhibited the production of ROS and RNS as efficiently as resveratrol. A significant gain in the cellular ROS/RNS inhibition capacity was observed for *N. lotus* extract resulting from UAE followed by DAX-8 MPR purification step (MPR) compared to extract obtained after conventional HRE (Figure 6b). Similar trends were observed in the production of TBARS, as evidenced by a significant reduction in lipid membrane peroxidation in yeast cells under oxidative stress condition in the presence of *N. lotus* extracts (Figure 6c). The best results were obtained with *N. lotus* extracts prepared after the UAE coupled with the DAX-8 MPR purification step. Mitochondria physiologically and continuously produced ROS and RNS as by-products of cellular metabolism. The production of ROS and RNS increases with age, stress or pollution as a direct consequence of redox cellular imbalances and could lead to the development of various degenerative diseases [65,66]. Flavonoids as strong natural antioxidants present in food may potentially counteract the negative effects of excessive ROS and RNS cellular production [64,67]. Yeast cells have been proposed as an excellent model for in vivo assessment of antioxidant capacity in relation to cellular oxidative stress [68]. It is an attractive and reliable eukaryotic model whose mechanisms of defense and adaptation to oxidative stress are well known and can be extrapolated to human cells with mechanisms that are more complex but well preserved with this model [69,70]. The present results confirm at a cellular level the trend observed using in vitro cell-free antioxidant assays, thus demonstrating the potential interest of the current extraction method in producing valuable antioxidant extracts from *N. lotus* stamen. The results also suggest that the antioxidant activity of the *N. lotus* extract is directly associated with flavonoid content and purity.

**Figure 7:**
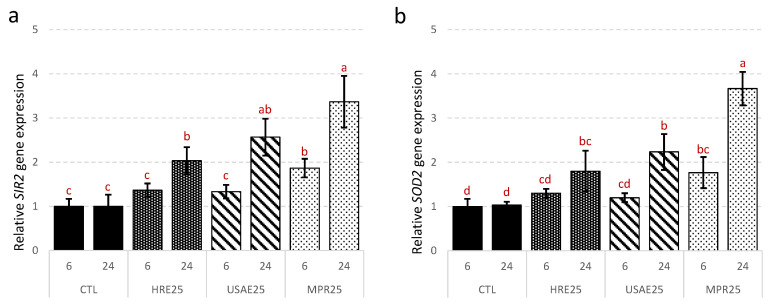
Effects of the different extracts from *N. lotus* stamen on *SIR2* (**a**) and *SOD2* (**b**) gene expression determined by RT-qPCR. Expression was normalized with *TUB1* gene. CTL are control (untreated, DMSO addition) yeast cells; HRE25 are yeast cells subjected to UV-induced oxidative stress in presence of 25 µg/mL of *N. lotus* extract obtained by HRE; UAE are yeast cells subjected to UV-induced oxidative stress in presence of 25 µg/mL of *N. lotus* extract obtained by UAE; MPR are yeast cells subjected to UV-induced oxidative stress in presence of 25 µg/mL of *N. lotus* extract obtained by UAE followed by DAX-8 MPR purification step. Values are means ± SD of three independent experiments. Different letters represent significant differences between the various extraction conditions (*p* < 0.05).

The next objective was to decode the molecular mechanism on which the cellular antioxidant activity of *N. lotus* extracts was based. The expression of two key genes involved in antioxidant defense was therefore monitored by qRT-PCR in yeast cells treated with the three *N. lotus* extracts (Figure 7).

The results suggested that the cellular antioxidant potential of *N. lotus* extracts could be the result of activation of gene expression of key antioxidant genes in yeast such as *SIR2* (silent information regulator 2) and *SOD2* (superoxide dismutase 2) (Figure 7a,b). Treatment with *N. lotus* extracts significantly increased the expression of *SIR2* and *SOD2* genes compared to control 24 h after treatment. Higher and faster stimulation of the expression of both gene was observed as soon as 6 h after treatment with *N. lotus* extract prepared after the UAE coupled with the DAX-8 MPR purification step.

*SIR2* encoded for a Nicotinamide Adenine Dinucleotide-dependent protein deacetylase and compelling evidence has linked its activity to oxidative stress response, in particular to ROS-driven mitochondrial-mediated response [66]. *SOD2* encodes for a mitochondrial Mn-SOD and plays a key role in the antioxidant response through effective ROS scavenging [71]. *SIR2* (aka SIRT1) has been proposed as an inducer of *SOD2* gene expression in various models [72,73]. The activation by different plant-derived natural products of both *SIR2* and *SOD2* gene expression has been associated with the increased antioxidant capacity [20,74,75,76]. The cellular antioxidant activity of the *N. lotus* extract may be linked to this ability to activate the expression of the key genes involved in the antioxidant response.

## 4. Conclusions

The stamens of the *N. lotus* medicinal plant are an alternative potential rich source of flavonoid antioxidants. This research employed current innovative green chemistry techniques, such as UAE, which are widely used for food, cosmetic and pharmaceutical industries together with MPR purification to enhance the antioxidant effect of flavonoids from its stamens. The optimal UAE condition is 90% (*v*/*v*) aqEtOH with 34.65 khz ultrasonic frequency and 46 min of extraction duration. Compared with the heat reflux extraction (HRE) conventional method, the significant 1.35-fold increase in total flavonoids content was obtained using optimized UAE conditions, jumping to a 2.80-fold increase when this UAE associated with MPR purification.

The results also indicated that UAE associated with MPR purification provided the best antioxidant potential of *N. lotus* stamens in both an in vitro (DPPH, ABTS and FRAP) and in cellulo yeast model comparing with the conventional HRE. In addition, the strongest cellular antioxidant activity (decreases of both ROS and RNS production as well as membrane lipid peroxidation (TBARS)) were observed in the extracts using UAE coupled with MPR purification. Furthermore, the best results of key antioxidant genes expression in eukaryotic yeast cell were detected using this innovative method. Indeed, the expression of key antioxidant genes such as *SIR2* and *SOD2* were also expressed at the highest level in yeast cell treated with the extract from UAE together with MPR purification.

According to these results, it is clearly seen that UAE associated with MPR purification step can enrich the antioxidant flavonoids potential of *N. lotus* stamens. This current discovery sheds new light on the applications of flavonoids from this medicinal plant in medical and pharmaceutical aspects for human health and well-being benefits. These results suggested that this innovative extract with high antioxidant capacity might be applied as effective antioxidants in the food industry. For the futures perspectives of *N. lotus* extract application, the enrichment in antioxidant flavonoids from this current research can be employed to test the potential of this stamen extract such as anticancer and the other pharmacological activities. Furthermore, this rich-antioxidant flavonoid extract may also be used as an alternative choice of an active ingredient for cosmetic product development.

## Figures and Tables

**Table 1:** Identity, code unit, coded levels and actual experimental values of each variable used for UAE of TFC from *N. lotus* stamens.

Variable	Code Unit	Coded Variable Levels		
		−1	0	+1
Ethanol concentration (% *v*/*v*) ^1^	X_1_	50	75	100
US frequency (kHz)	X_2_	0	22.5	45
Extraction duration (min)	X_3_	20	40	60

^1^ Percentage of ethanol (analytical grade) concentration in mixture with ultrapure water (HPLC grade).

**Table 2:** Characteristics of the MPR used in the present study.

Resins	Surface Area (m^2^/g)	Particle Diameter (µm)	Average Pore Diameter (Å)	Matrix Type	Polarity
**XAD-2**	300	560–710	90	Styrene-divinyl-benzene macroreticulate	Hydrophobic
**XAD-4**	750	250–840	100	Styrene-divinyl-benzene	Hydrophobic
**XAD-16**	800	560–710	200	Styrene-divinyl-benzene	Hydrophobic
**XAD-7**	380	560–710	300–400	Acrylic	Moderately polar
**DAX-8**	140	250–420	225	Acrylic ester	Moderately polar

**Table 3:** Results of full factorial design experiments for the UAE of TFC from *N. lotus* stamens.

Run ID	Run Order	X_1_	X_2_	X_3_	Experimental TFC (mg/g DW)	Predicted TPC (mg/g DW)
Obs1	4	−1	−1	−1	29.68 ± 2.75	33.75
Obs2	23	0	−1	−1	132.73 ± 6.96	134.83
Obs3	17	+1	−1	−1	142.43 ± 3.71	151.11
Obs4	19	−1	0	−1	67.69 ± 2.79	60.99
Obs5	24	0	0	−1	175.81 ± 4.95	177.81
Obs6	22	+1	0	−1	208.71 ± 5.93	209.80
Obs7	16	−1	+1	−1	47.24 ± 2.02	42.86
Obs8	6	0	+1	−1	180.17 ± 2.90	175.40
Obs9	14	+1	+1	−1	225.21 ± 3.56	223.13
Obs10	27	−1	−1	0	47.67 ± 3.37	43.61
Obs11	11	0	−1	0	158.32 ± 3.64	148.30
Obs12	1	+1	−1	0	180.84 ± 4.82	168.18
Obs13	8	−1	0	0	58.67 ± 3.74	67.08
Obs14	20	0	0	0	188.82 ± 2.52	187.51
Obs15	25	+1	0	0	228.89 ± 5.53	223.11
Obs16	5	−1	+1	0	31.71 ± 4.02	45.19
Obs17	2	0	+1	0	176.27 ± 3.51	181.34
Obs18	10	+1	+1	0	225.81 ±5.99	232.67
Obs19	7	−1	−1	+1	39.86 ± 2.85	40.03
Obs-20	18	0	−1	+1	144.12 ± 2.72	148.33
Obs21	12	+1	−1	+1	164.30 ± 3.23	171.82
Obs22	21	−1	0	+1	61.11 ± 1.30	59.74
Obs23	13	0	0	+1	178.45 ± 8.61	183.77
Obs24	26	+1	0	+1	224.66 ± 5.50	222.99
Obs25	9	−1	+1	+1	43.71 ± 2.73	34.08
Obs26	15	0	+1	+1	176.45 ± 7.33	173.84
Obs27	3	+1	+1	+1	230.74 ± 5.97	228.78

Values are the means ± SD of three independent replicates.

**Table 4:** Statistical analysis of the regression coefficients of UAE of TFC from *N. lotus* stamens.

Source	Value	SD	*t*	*P* > |*t*|
Constant	187.507	3.984	47.064	<0.0001 ***
X_1_	78.015	1.844	42.301	<0.0001 ***
X_2_	16.520	1.844	8.957	<0.0001 ***
X_3_	2.984	1.844	1.618	0.124
X_1_^2^	−42.408	3.194	−13.276	<0.0001 ***
X_2_^2^	−22.687	3.194	−7.102	<0.0001 ***
X_3_^2^	−6.717	3.194	−2.103	0.051
X_1_X_2_	15.728	2.259	6.963	<0.0001 ***
X_1_X_3_	3.608	2.259	1.597	0.129
X_2_X_3_	−3.764	2.259	−1.666	0.114

SD standard deviation; *** significant *p* < 0.001.

**Table 5:** ANOVA of the predicted model for used for UAE of TFC from *N. lotus* stamens.

Source	Sum of Square	df	Mean of Square	*F*-Value	*p*-Value
Model	132,069.810	9	14,674.423	239.686	<0.0001 ***
Lack of fit	1040.801	17	61.224	-	-
Residual	1036.980	17	60.999	-	-
Pure Error	3.822	0	-	-	-
Cor. Total	133,110.611	26	-	-	-
R^2^	0.992				
R^2^ adj	0.988				
CV %	0.979				

df: degree of freedom; Cor. Total: corrected total; R^2^: determination coefficient; R^2^ adj: adjusted R^2^; CV variation coefficient value; *** significant *p* < 0.001.

**Table 6:** Relative quantification of the different flavonoid glucosides in the *N. lotus* stamen extracts.

Peak Number	Tentative Identification	HRE	USAE	MPR
1	Myr 3-O-Gal	21.1 ± 1.3	28.3 ± 1.1	76.9 ± 3.8
2	Myr 3′-O-Xyl	34.4 ± 1.7	45.7 ± 2.1	129.3 ± 6.7
3	Que-3-O-Rha	62.1 ± 3.1	86.3 ± 1.4	244.2 ± 5.1
4	CNar-2″-O-Gal	16.4 ± 1.3	22.9 ± 1.3	64.7 ± 3.6
5	Kae-3-O-Gal	54.7 ± 2.3	73.4 ± 3.2	200.2 ± 5.4
6	Que-3′-O-Xyl	29.1 ± 1.5	41.2 ± 2.1	117.2 ± 2.4
7	Iso-7-O-Gal	24.8 ± 1.6	33.6 ± 1.7	90.4 ± 3.3
8	Iso-7-O-Xyl	41.4 ± 2.4	55.4 ± 2.3	144.2 ± 5.7
9	Iso-3-O-Xyl	13.2 ± 1.4	19.7 ± 1.1	56.6 ± 1.7

Expressed in absorbance unit per g DW; HRE: *N. lotus* extract obtained by HRE; USAE: *N. lotus* extract obtained by USAE; MPR: *N. lotus* extract obtained by USAE followed by DAX-8 MPR purification step.

**Table 7:** TFC and in vitro cell-free antioxidant activity of the different extracts from *N. lotus* stamen.

Sample	TFC (mg/g DW)	DPPH (µmol TEAC ^1^/g)	ABTS (µmol TEAC ^1^/g)	FRAP (µmol TEAC ^1^/g)
**HRE**	169.64 ± 9.86 ^c^	2134.37 ± 130.84 ^c^	2488.95 ± 344.45 ^c^	1270.71 ± 65.53 ^a^
**UAE**	235.45 ± 5.44 ^b^	3167.85 ± 342.64 ^b^	3218.56 ± 234.90 ^b^	2405.25 ± 315.86 ^b^
**MPR**	475.42 ± 16.61 ^a^	4749.67 ± 166.05 ^a^	4710.67 ± 155.64 ^a^	3601.74 ± 126.26 ^a^
**BHT**	-	3059.07 ± 401.32 ^b^	4378.12 ± 106.62 ^a^	1724.12 ± 161.27 ^b^

^1^ TEAC: Trolox C equivalent antioxidant capacity. HRE: heat reflux extraction; UAE: ultrasound assisted extraction; MPR: UAE following by DAX-8 MPR purification step. Values are means ± SD of three independent experiments. Different letters represent significant differences between the various extraction conditions (*p* < 0.05).

## Supplementary Materials

The following are available online at https://www.mdpi.com/2076-3921/9/7/576/s1, Figure S1: Biplot representation of the linear relation between predicted vs. measured TFC in the 27 *N. lotus* sample extracts; Figure S2: Normalized coefficients representation with the 95% CI for the proposed polynomial model. Table S1: Conductivity and total reducing sugar contents in the different extracts from *N. lotus* stamen; Table S2: Characteristic and tentative identification of flavonoids from *N. lotus* stamen extract.
