# Supplementary material for: Enrichment in Antioxidant Flavonoids of Stamen Extracts from Nymphaea lotus L. Using Ultrasonic-Assisted Extraction and Macroporous Resin Adsorption
Source: Antioxidants (Basel). 2020 Jul 2;9(7):576. doi: 10.3390/antiox9070576 (PMC7402147; doi:10.3390/antiox9070576)
Supplement: Supplementary file 1 [file antioxidants-09-00576-s001.pdf]

## SUPPLEMENTARY MATERIALS

---

# Enrichment in Antioxidant Flavonoids of Stamen Extracts from *Nymphaea lotus* L. using Ultrasonic-Assisted Extraction and Macroporous Resin Adsorption

**Duangjai Tungmunnithum [1,2,3,*], Samantha Drouet [2,3], Atul Kabra [4], and Christophe Hano [2,3,*]**

[1] Department of Pharmaceutical Botany, Faculty of Pharmacy, Mahidol University, Bangkok 10400, Thailand; duangjai.tun@mahidol.ac.th (D.T.);
[2] Laboratoire de Biologie des Ligneux et des Grandes Cultures, INRA USC1328, University of Orleans, 45067 Orléans CEDEX 2, France; samantha.drouet@univ-orleans.fr (S.D.)
[3] Bioactifs et Cosmetiques, CNRS GDR 3711 Orleans, 45067 Orléans CEDEX 2, France
[4] School of Pharmacy, Raffles University, Neemrana 301705, Alwar, Rajasthan, India; atul.kbr@gmail.com
* Correspondence: duangjai.tun@mahidol.ac.th (D.T.); hano@univ-orleans.fr (C.H.); Tel.: +66-264-486-96 (D.T.); +33-237-309-753 (C.H.)

## CONTENT:

**Figure S1:** Biplot representation of the linear relation between predicted vs. measured TFC in the 27 *N. lotus* sample extracts – page 2.

**Figure S2:** Normalized coefficients plot with the 95% CI interval for the proposed polynomial model – page 3.

**Table S1:** Conductivity and total reducing sugar contents in the different extracts from *N. lotus* stamen – page 4.

**Table S2:** Characteristic and tentative identification of flavonoids from *N. lotus* stamen extract – page 5.

**Figure S1:** Biplot representation of the linear relation between predicted vs. measured TFC in the 27 *N. lotus* sample extracts.

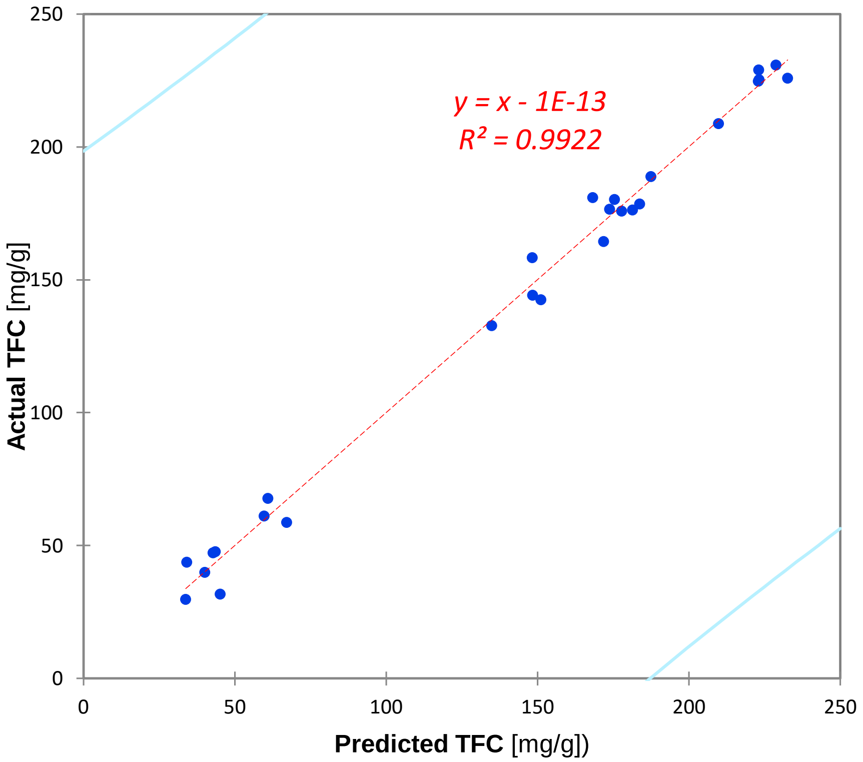


Light blue lines represented 95% confidence interval.

**Figure S2:** Normalized coefficients representation with the 95% CI for the proposed polynomial model.

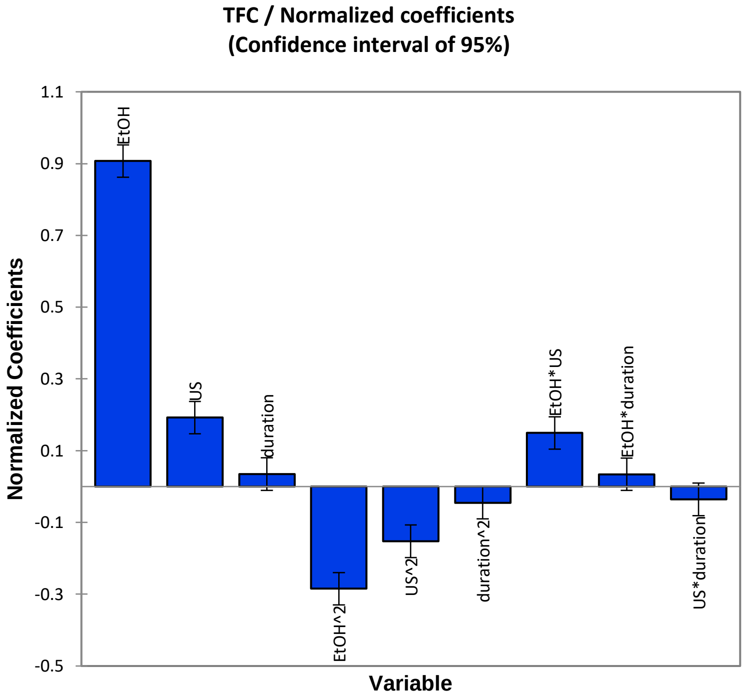

**Table S1:** Conductivity and total reducing sugar contents in the different extracts from *N. lotus* stamen.

| **Extract** | **Conductivity** (μS/cm) | **Reducing sugar content** (AU/mL[1]) |
|---|---|---|
| HRE | 0.039 ± 0.005 | 0.056 ± 0.011 |
| USAE | 0.122 ± 0.010 | 0.065 ± 0.010 |
| MPR | 0.044 ± 0.012 | 0.007 ± 0.006 |

[1] AU/mL: expressed in absorbance unit per mL of extract; HRE: *N. lotus* extract obtained by HRE; USAE: *N. lotus* extract obtained by USAE; MPR: *N. lotus* extract obtained by USAE followed by DAX-8 MPR purification step.

**Table S2:** Characteristic and tentative identification of flavonoids from *N. lotus* stamen extract

| Peak number | Retention time (min) | λmax (nm) | $[M\text{-}H]^-$ | Tentative identification | Commercial standard | Reference |
|---|---|---|---|---|---|---|
| 1 | 29.11 | 263,349 | 479 | Myr 3-O-Gal | + (ES) | Zhu et al., 2012 |
| 2 | 31.67 | 254,305,366 | 449 | Myr 3′-O-Xyl | - | Zhu et al., 2012 |
| 3 | 32.26 | 257,348 | 447 | Que-3-O-Rha | + (SA) | Zhu et al., 2012; Yin et al., 2015 |
| 4 | 32.87 | 250,366 | 433 | CNar-2′′-O-Gal | - | Zhu et al., 2012; Yin et al., 2015 |
| 5 | 33.52 | 265,343 | 447 | Kae-3-O-Gal | + (SA) | Zhu et al., 2012 |
| 6 | 33.91 | 254,366 | 433 | Que-3′-O-Xyl | + (SA) | Zhu et al., 2012 |
| 7 | 34.71 | 268,350 | 477 | Iso-7-O-Gal | - | Zhu et al., 2012; Yin et al., 2015 |
| 8 | 35.51 | 252,268,352 | 447 | Iso-7-O-Xyl | - | Zhu et al., 2012; Yin et al., 2015 |
| 9 | 36.57 | 265, 343 | 447 | Iso-3-O-Xyl | - | Yin et al., 2015 |

Myr: myricetin; Que: quercetin; CNar: chalcononaringenin; Kae: kaempferol; Iso: isorhamnetin; Gal: galactoside; Xyl: xyloside; Rha: rhamnoside. Standards: + = available; - = not available; ES = commercial standard from Extrasynthese (Genay, France); SA = commercial standard from Sigma-Aldrich (Saint-Quentin Fallavier, France); Commercial standards from Sigma-Aldrich (Saint Quentin Fallavier, France): Que-3-O-Rha, phyproof® Reference Substance, with purity ≥95.0% (HPLC); Que-3′-O-Xyl with purity ≥97.0% (HPLC); Kae-3-O-Gal with purity ≥90% (LC/MS-ELSD); Commercial standard from Extrasynthese (Genay, France): Myr 3-O-Gal with purity ≥99%(HPLC). References: Zhu et al., 2012 (reference 14, main manuscript): Zhu, M., Zheng, X., Shu, Q., Li, H., Zhong, P., Zhang, H., ... & Wang, L. (2012). Relationship between the composition of flavonoids and flower colors variation in tropical water lily (Nymphaea) cultivars. *PloS one*, *7*(4); Yin et al., 2015 (reference 4, main manuscript): Yin, D. D., Yuan, R. Y., Wu, Q., Li, S. S., Shao, S., Xu, Y. J., ... & Wang, L. S. (2015). Assessment of flavonoids and volatile compounds in tea infusions of water lily flowers and their antioxidant activities. *Food chemistry*, *187*, 20-28.
